# Insights into patient characteristics and documentation of the use of sedative-hypnotic/anxiolytics in primary care: a retrospective chart review study

**DOI:** 10.1186/s12875-022-01724-9

**Published:** 2022-05-10

**Authors:** Kiana Gozda, Joyce Leung, Lindsay Baum, Alexander Singer, Gerald Konrad, Diana E. McMillan, Jamie Falk, Leanne Kosowan, Christine Leong

**Affiliations:** 1 College of Pharmacy, Rady Faculty of Health Sciences, University of Manitoba, Winnipeg, Manitoba Canada; 2 Faculty of Science, University of Manitoba, Winnipeg, Manitoba Canada; 3 Department of Family Medicine, Rady Faculty of Health Sciences, Max Rady College of Medicine, University of Manitoba, Winnipeg, Manitoba Canada; 4 College of Nursing, Rady Faculty of Health Sciences, University of Manitoba, Winnipeg, Manitoba Canada; 5 Deparment of Psychiatry, Rady Faculty of Health Sciences, Max Rady College of Medicine, University of Manitoba, Winnipeg, Manitoba Canada

## Abstract

**Background:**

Despite the known safety risks of long-term use of sedative-hypnotic/anxiolytic medications, there has been limited guidance for the safe and effective use of their chronic use in a primary care clinic setting. Understanding the characteristics of patients who receive sedative-hypnotic/anxiolytic medication and the clinical documentation process in primary care is the first step towards understanding the nature of the problem and will help inform future strategies for clinical research and practice.

**Objectives:**

Characterize patients who received a sedative-hypnotic/anxiolytic prescription in primary care, and (2) gain an understanding of the clinical documentation of sedative-hypnotic/anxiolytic indication and monitoring in electronic medical records (EMR).

**Methods:**

A random selection of patients who received a prescription for a benzodiazepine or Z-drug hypnotic between January 2014 and August 2016 from four primary care clinics in Winnipeg were included. Data was collected retrospectively using the EMR (Accuro®). Patient variables recorded included sex, age, comorbidities, medications, smoking status, and alcohol status. Treatment variables included drug type, indication, pattern of use, dose, adverse events, psychosocial intervention, tapering attempts, social support, life stressor, and monitoring parameters for sedative-hypnotic use. Demographic and clinical characteristics were described using descriptive statistics.

**Results:**

Records from a sample of 200 primary care patients prescribed sedative-hypnotic/anxiolytics were analyzed (mean age 55.8 years old, 61.5% ≥ 65 years old, 61.0% female). Long-term chronic use (≥ 1 year) of a sedative-hypnotic/anxiolytic agent was observed in 29.5% of the sample. Zopiclone (30.7%) and lorazepam (28.7%) were the most common agents prescribed. Only 9.5% of patients had documentation of a past tapering attempt of their sedative-hypnotic/anxiolytic. The most common indications for sedative-hypnotic/anxiolytic use recorded were anxiety (33.0%) and sleep (18.0%), but indication was undetermined for 57.0% of patients. Depression (33.5%) and falls (18.5%) were reported by patients after the initiation of these agents.

**Conclusions:**

A higher proportion of females and users 65 years and older received a prescription for a sedative-hypnotic/anxiolytic, consistent with previous studies on sedative-hypnotic use. We found inconsistencies in the documentation surrounding sedative-hypnotic/anxiolytic use. The indication for their use was unclear in a large number of patients. These findings will help us understand the state of the problem in primary care and inform future strategies for clinical research.

**Supplementary Information:**

The online version contains supplementary material available at 10.1186/s12875-022-01724-9.

## Introduction

Benzodiazepines and Z-hypnotics (such as zopiclone and zolpidem) are commonly prescribed for sleep disorders [1–8]. Benzodiazepines are also widely used to treat anxiety, and as adjunct treatment for depression, and pain management [1–5, 8]. Although less frequent, benzodiazepines can also be used for treatment of seizures and muscle spasticity [1–5]. Approximately 10% of Canadians have reported using prescription sedative-hypnotic/anxiolytics, with the highest rates of use among females and the elderly [1, 2, 6, 7]. In Manitoba, the prevalence of benzodiazepines use remained steady at about 6.1% between 1996 to 2012, but the prevalence of Z-hypnotic use increased 3.4 fold from 1.1% to 3.7% [9]. While the use of these agents is recommended to be short term and limited to a few weeks, some individuals remain on these agents for years or even decades [1–5, 10]. A Manitoba study found 3.8% of all individuals who started a benzodiazepine or Z-hypnotic become sustained users of these agents for at least two years with the average years of continuous use being 4.4 years [10]. A study of 64 primary care clinics in Quebec found benzodiazepines were used by 22.6% with anxiety disorder specifically and 88.4% used these agents, both regular and as-needed dosing, for more than 12 weeks [11]. Another study by Steinman et al. have reported 40% of individuals, with 33% of individuals 18–64 years and 59% of individuals 65 years and older, using these agents for approximately 6 months or greater [12]. In a cross-sectional population-based study by Johnson et al., 11.5% of non-care home residents and 28.4% of care home residents in Scotland 65 years and older were receiving a benzodiazepine or Z-drug [13]. Pinsker et al. identified 36.2% of 257 primary care elderly patients (mean age 72 years old) were receiving a benzodiazepine for one or more years [14].

Chronic sedative-hypnotic use has been linked to interference with daytime functioning, motor vehicle accidents, fall risk and injury, cognitive impairment, and physical dependence [1, 4–10, 15–21]. Withdrawal symptoms include anxiety, depression, agitation, nausea, vomiting, hallucination, hypersensitivity to stimuli, perceptual distortions, depersonalizations, and seizures [1, 4, 5, 15, 16]. The risk of dependence, withdrawal and rebound symptoms, and abuse has been associated with higher doses, longer duration of use (e.g., over a year), shorter acting agents (e.g., triazolam), and in patients with a history of psychiatric illness [17]. As a result, discontinuation protocols must be done slowly with careful monitoring to minimize the effects of withdrawal.

There is an evidence gap to direct the management of patients who continue to experience acute insomnia or anxiety beyond the recommended duration of use of sedative-hypnotic/anxiolytics in primary care. Simple interventions, including clinician letters, self-help information, and consultation with a general practitioner aimed at informing patients of the risk of chronic benzodiazepines use and the benefits of discontinuation, have been found to be effective at promoting discontinuation [4, 5]. However, reluctance or inability of the patient to stop their sedative-hypnotic/anxiolytic is still a prevalent issue in primary care [22–24]. Furthermore, evidence for the long-term efficacy and safety of de-prescribing sedative-hypnotic/anxiolytics has major gaps [25]. The best approach to monitoring the use and discontinuation of these agents is still under investigation. In the absence of sufficient evidence to direct the appropriate management of patients who are on a sedative-hypnotic/anxiolytic in primary care, understanding the use and monitoring of these agents in primary care will allow for further investigations on interventions, such as tapering protocols, and their effectiveness.

Primary care providers are in an opportune position for recognizing non-optimal sedative-hypnotic/anxiolytic use and to aid patients in the safe discontinuation of such agents. Previous studies have also found that primary care physicians (more than 80%) rather than psychiatrists (approximately 15%) write most of the new benzodiazepine and Z-hypnotic prescriptions [9, 23, 24]. In light of the known long-term risks associated with the chronic use of these agents and the challenge of discontinuing these agents after long-term therapy, we aim to: characterize patients who received a sedative-hypnotic/anxiolytic prescription in primary care, and gain an understanding of the clinical documentation of sedative-hypnotic/anxiolytic indication and pattern of use in electronic medical records (EMR). These findings will help us understand the state of the problem in primary care and inform future strategies for clinical research.

## Methods

### Study design

This is a retrospective chart review using data from the EMR at four primary care clinics.

### Study population and setting

A random sample, using a random generator, of 200 electronic medical records of patients who received a prescription for a sedative-hypnotic/anxiolytic and were an active patient between January 2014 and August 2016 at four primary care clinics in Winnipeg, Manitoba, Canada were included. Two of the clinics are interprofessional teaching clinics. The other two clinics are interprofessional clinics that serve patients with complex health needs. Sample size was determined based on previous literature that we may expect 40% of patients would have received a “sustained” (> 6 months) prescription for a sedative-hypnotic/anxiolytic [12]. This percentage was used as it reflects our population receiving a sedative-hypnotic/anxiolytic not restricted by indication or age, or requiring a duration of greater than 2 years to be considered long-term use used in other studies. Sedative-hypnotic/anxiolytic agents included specific benzodiazepines (alprazolam, bromazepam, clonazepam, diazepam, flurazepam, nitrazepam, oxazepam, temazepam, triazolam) and “Z” hypnotics (zolpidem, zopiclone).

### Data collection

All information was retrieved from the medical history band, medication band, and clinical encounter notes from the EMR (Supplementary Table 1). Information on the sedative-hypnotic use status, pattern of use, duration of use, frequency of use, co-medications, date of first and last prescription, and external medication use was obtained from the medication band of the EMR. Pattern of use was categorized into six categories: (1) Short-term (≤ 8 weeks), short-term regular (> 8 weeks to < 3 months), intermittent regular (≥ 3 months to < 1 year), long-term regular (≥ 1 year), intermittent (variable duration of use with > 1 month breaks between each use), and other (e.g., not specified). Regular use was defined as continued use with ≤ 1 month gap in days supply between prescription renewals. Sedative-hypnotic/anxiolytic use status was defined as new (active prescription with the first prescription started in the last 365 days), chronic (active prescription with the first prescription started more than 365 days ago), and past use (prescription is inactive) as identified in the medication band of the EMR. A prescription was considered “inactive” if the prescription was manually inactivated by the prescriber on the EMR or the prescription was automatically inactivated by the EMR as a result of the prescription being expired (i.e., days since the prescription was written exceeded the days supply provided on the prescription). Medical conditions (categorized by ICD-9-CM category), smoking status, and alcohol use were obtained from the medical history band of the EMR. Encounter notes were reviewed for documentation of psychosocial / behavioural intervention use (including cognitive behavioural therapy, mindfulness-based cognitive behavioural therapy, exposure-based intervention, relaxation training, sleep hygiene, meditation) [26, 27], social support, and life stressors at any time before or after sedative-hypnotic/anxiolytic use. Encounter notes were also reviewed after sedative-hypnotic/anxiolytic initiation for tapering attempts, adverse events reported after sedative-hypnotic/anxiolytic initiation, and monitoring scales or tests conducted after sedative-hypnotic/anxiolytic initiation. Age and sex were also retrieved from demographic band. The data collection form was piloted in July to August 2017 for 40 patients (10 at each site) prior to data collection to ensure all pertinent information was being captured. Data collection for the remaining patients occurred from May to August 2018. Three research assistants (LB, KG, JL) were trained and carried out the data collection.

**Table 1:** Demographics and characteristics of primary care patients who were prescribed a sedative-hypnotic/anxiolytic (*n* = 200)

Characteristic	Value
**Age (years)**	
Mean	55.8
Median (range)	57 (20–95)
65 + **(n, %)**	123 (61.5)
**Female sex** (n,%)	122 (61.0)
**Smoking status** (n,%)	
Current	50 (25.0)
Past	24 (12.0)
None	50 (25.0)
Not recorded	76 (38.0)
**Alcohol use** (drinks/week; n,%)	
none	39 (19.5)
< 7	37 (18.5)
7–14	7 (3.5)
> 14	6 (3.0)
Not recorded	111 (55.5)
**Psychosocial intervention used** (n,%)	87 (43.5)
**Past tapering attempt** (n,%)	19 (9.5)
**Pattern of use** (n,%)	
Short term use (≤ 8 weeks)	57 (28.5)
Intermediate regular use **(> 8 weeks** to < 1 year)	76 (38.0)
Long-term regular use **(≥ 1 year)**	59 (29.5)
Intermittent use (variable duration of use with > 1 month break between use)	45 (22.5)
Other (e.g., SIG not detailed, active external medication)	11 (5.5)
**Sedative-hypnotic/anxiolytic use status** (n,%)	
New (prescription is active and first prescribed ≤ 1 year ago)	15 (7.5)
Sustained (prescription is active and first prescribed > 1 year ago)	66 (33.0)
Past (prescription is inactive)	117 (58.5)
**Medical conditions** (n,%)	
Cardiovascular	178 (89.0)
Mental health	132 (66.0)
Endocrine	71 (35.5)
Gastro-intestinal	70 (35.0)
Musculoskeletal	69 (34.5)
Genitourinary	69 (34.5)
Hepatobiliary	49 (24.5)
Respiratory	35 (17.5)
Cancer	35 (17.5)
Pain	31 (15.5)
Infectious Disease	19 (9.5)
Dermatological	18 (9.0)
Neurology	16 (8.0)

### Data analysis

All data were reported using descriptive statistics. Mean and proportions were reported for demographic and clinical characteristics. Distribution of sedative-hypnotic/anxiolytic type was reported as proportion. Indication of use, adverse events, and monitoring scales were reported as frequency. All data were analyzed using Microsoft Excel® (Microsoft Corporation, Redmond, WA).

### Patient engagement

The study investigators actively collaborated with a patient and caregiver/family representative advisory group as part of the research team, who provided input in all aspects of the research study, ensuring the research priorities and question, methods used, interpretation and dissemination of findings, and the development of educational tools were appropriate. These representatives contributed to ensure the study and results will be meaningful to patients and to the development of a long-term action plan for patients to be educated about the appropriate use of these agents.

### Ethics

This study was approved by the University of Manitoba Human Research Ethics Board (HREB) (Ethics # HS20239 (H2016:400), and the Winnipeg Regional Health Authority Research Review Committee. All methods were carried out in accordance with relevant guidelines and regulations. Informed consent was waived by the University of Manitoba HREB for this study.

## Results

Among the sample of 200 primary care patients meeting study inclusion, the mean age was 55.8 years (61.5% were ≥ 65 years) and 61.0% were female (Table 1). With respect to pattern of use, most patients were prescribed sedative-hypnotic/anxiolytic medication regularly for greater than eight weeks but less than a year (38.0%). Long-term (≥ 1 year) regular use of a sedative-hypnotic/anxiolytic agent was observed in 29.5% and short-term use (≤ 8 weeks) was found in 28.5% of the sample. In terms of sedative-hypnotic/anxiolytic use status, more than half of patients had an inactive prescription (58.5%) and a third of patients had sustained use (prescription is active and first prescribed more than one year ago). Eighty-one patients had an active prescription for a sedative-hypnotic/anxiolytic, of which 15 (or 18.5% of active prescriptions) were prescribed their first sedative-hypnotic/anxiolytic less than one year ago and 66 (or 33%) were prescribed their first prescription over a year ago. Only 9.5% of patients had documentation of a past tapering attempt of their sedative-hypnotic/anxiolytic and 43.5% had documentation of using a psychosocial intervention. Smoking status and alcohol use were not documented in the chart in 38.0% and 55.5%, respectively.

**Fig. 1:**
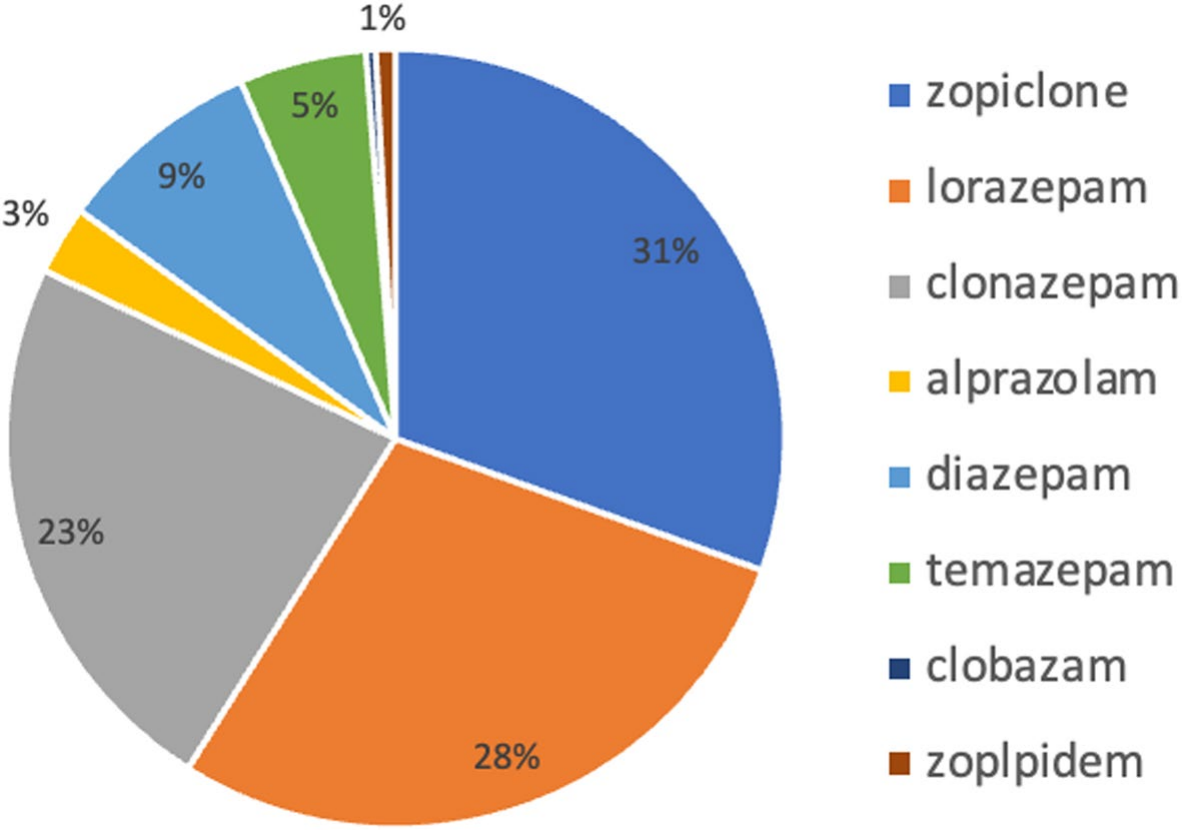
Distribution of type of sedative-hypnotic/anxiolytic prescribed in primary care. *246 records of sedative-hypnotic/anxiolytic use recorded for 200 patients. There were 44 patients who had more than one different type of sedative-hypnotic/anxiolytic recorded in their chart

**Fig. 2:**
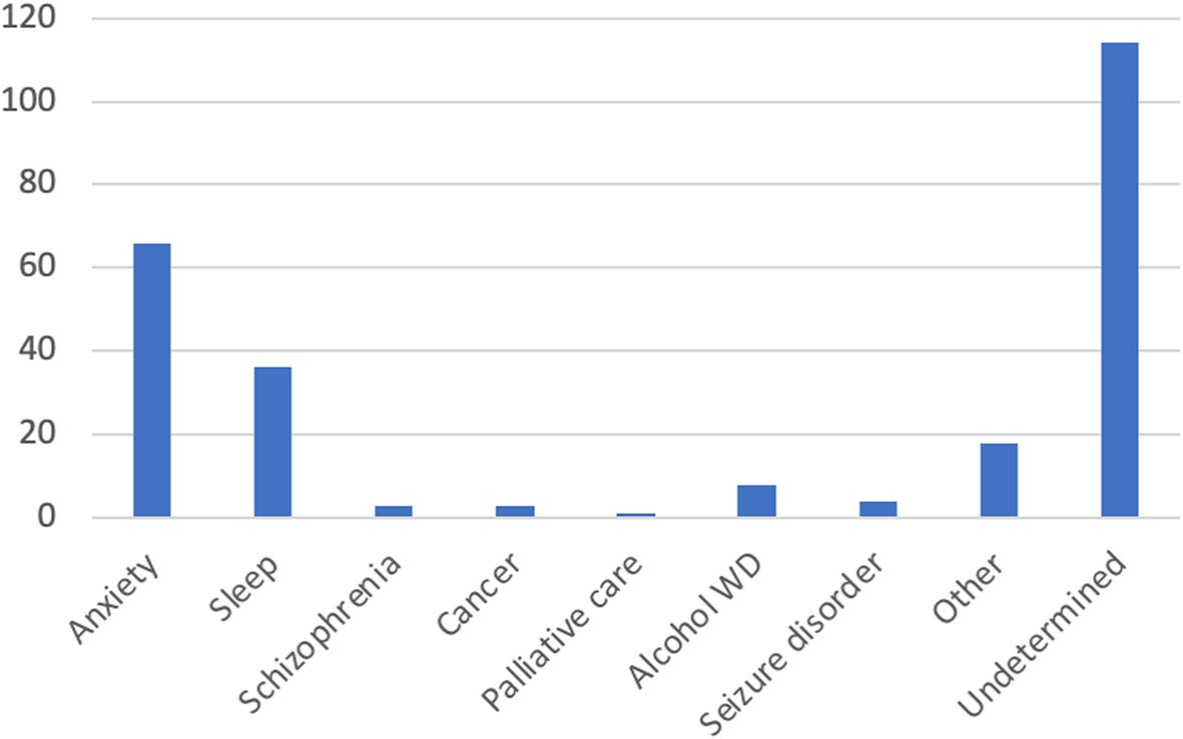
Frequency of indications recorded for sedative-hypnotic/anxiolytic use. *Other: panic attacks, withdrawal prophylaxis, post traumatic stress disorder, use prior to medical procedure, bipolar disorder, depression, plane phobia, stress, mood, shoulder tendinopathy, gastrointestinal scope, Alzheimer’s, uncontrollable anger, assist tapering, restless leg syndrome

**Fig. 3:**
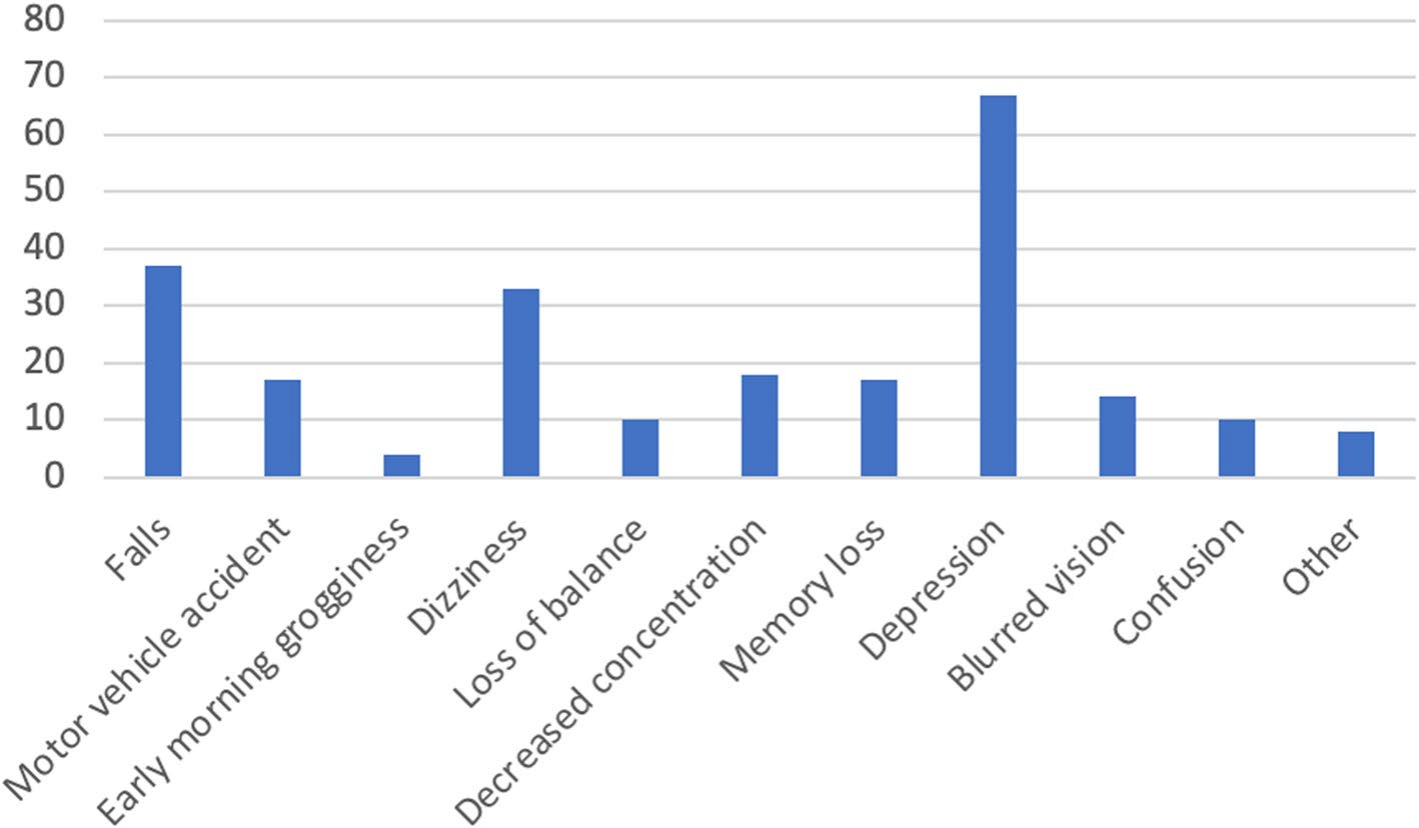
Frequency of adverse events experienced by patients after sedative-hypnotic/anxiolytic initiation. *Other: suicidal ideation, polydrug overdose, nightmares

Zopiclone (30.4%) was the most common agent prescribed followed by lorazepam (28.4%) and clonazepam (23.2%) (Fig. 1). There were 22% (*n* = 44) of patients who had more than one active sedative-hypnotic/anxiolytic types recorded in their EMR. The most common indications for sedative-hypnotic/anxiolytic use recorded were anxiety (33.0%) and sleep (18.0%), but indication was undetermined for 57.0% of patients (Fig. 2). Depression (33.5%), falls (18.5%), and dizziness (16.5%) were the most commonly recorded adverse events experienced by patients after the initiation of these agents (Fig. 3).

## Discussion

This study found that in a random sample of primary care patients with an active prescription for a sedative-hypnotic/anxiolytic agent, more than two-thirds (67.5%) of patients have regularly used it for greater than eight weeks, exceeding the duration of use recommended by clinical practice guidelines for insomnia (2–4 weeks) [28] and anxiety (8 weeks) [26]. Moreover, among the 40.9% with an active prescription, only 18.5% received their first prescription for a sedative-hypnotic/anxiolytic within the prior year, and 81.5% of patients received their first prescription over a year ago, supporting a concerning conclusion that nearly a third of the patients sampled have been prescribed sedative-hypnotic agent use on a chronic long-term basis. These rates are similar to previous studies by Steinman et al. [12] and Pinsker et al. [14] in primary care. Previous studies reporting on the prevalence of long-term benzodiazepine or z-drug use have been derived from administrative data or self-reported surveys. One study using outpatient pharmacy claims data reported sustained benzodiazepine or z-drug use (defined as duration of two or more years) of 3.8% among all individuals who received a prescription in Manitoba from 1996 to 2008 [10]. Determination of indication was not possible in this study but 15.2% had a diagnosis of anxiety disorder, 43.1% had depression, and 42.1% had a seizure disorder. Another study in France using health insurance data have found the prevalence of continuous long-term benzodiazepine use (defined as greater than 12 weeks) to be 2.8% for men and 3.8% for women [29]. A population-based cohort study conducted in Finland reported 39.4% of incident benzodiazepine users became long-term users (defined as duration of greater than 180 days) [30]. In this study by Airagnes et al., zopiclone was the most common drug used (31.9% in younger population, 39.6% in older population). The most common indication for the benzodiazepine was anxiety and depression (unadjusted estimated benzodiazepine visit rate 33.5%, 95% CI 28.8–38.6) and insomnia (25.6%, 15.3–39.6) [30]. The 12-month prevalence of long-term continuous benzodiazepine use among 1423 community-dwelling older adults (65 years and older) living in Quebec who participated in the Canadian Study of Health and Aging (a study designed to study the prevalence of dementia in Canada) was 19.8% [31].

We also identified that less than 10% of the study group had documentation of a history of a past tapering attempt of their sedative-hypnotic/anxiolytic, and less than half of patients had documentation of using a psychosocial / behavioural intervention. A scoping review on benzodiazepine deprescribing found 41% (*n* = 30) out of 74 original studies found a non-significant effect of the intervention for stopping benzodiazepines and/or z-drugs [32]. A positive effect was found in 47% (*n* = 35) of studies and 12% (*n* = 9) did not provide enough data to clearly assess the direction of effect. Psychological therapies to facilitate discontinuation were studied in 10 studies (14%) with 60% of these trials using CBT. Other strategies used included anxiety management, stress management, and psychotherapy. Further research into improving the acceptance and feasibility of non-drug interventions for assisting in benzodiazepine and z-drug discontinuation is needed.

Moreover, the indication of use was undetermined for more than half of patients. These findings indicate that there are inconsistencies in both documenting the rationale for sedative-hypnotic/anxiolytic use and in recording a plan for monitoring and tapering these agents despite of our knowledge on the minimal benefit of these agents beyond four to eight weeks of use [26, 28]. These findings may also suggest that there are difficulties in discontinuing these agents and/or implementing psychosocial / behavioural interventions for those who continue to experience insomnia and anxiety beyond the recommended duration of use. Clinical trials evaluating benzodiazepine discontinuation strategies have reported a success rate ranging from 32 to 59% [33–37], which speaks to the need to develop more effective strategies.

The use of a benzodiazepine or Z-drug for a period longer than that recommended by guidelines that was observed in our study could be explained by the length of time it takes to find a safe and effective long-term therapy for anxiety and/or insomnia. For instance, selective serotonin reuptake inhibitors (SSRIs) are considered first-line pharmacotherapy for many types of anxiety disorders, however, these agents often take four to six weeks to become effective [26]. In some cases, patients may not respond to their first SSRI and would need to switch to a different SSRI, which may prolong a time an individual would require a benzodiazepine. Likewise, cognitive behavioral therapy (CBT) is considered first-line for the treatment of insomnia and evidence has demonstrated its effects are longer lasting than drug therapy with a benzodiazepine receptor agonist [28]. However, access to in-person publicly funded CBT is limited to waitlists of weeks to months, and it often requires major behavioral changes of the individual, which can take time for CBT to become effective. As a result, documentation of initiation of first-line therapies for anxiety and/or insomnia and following up on the efficacy, safety (e.g., side effects, potential drug interactions), and convenience (e.g., cost, coverage, adherence) of the therapy for the patient would be important to address concerns early and to re-visit the need for continued benzodiazepine receptor agonist therapy or to begin their taper. With respect to tapering, previous literature have recommended tapering benzodiazepines within a six month period to minimize the effect of the withdrawal process from becoming a morbid focus for a prolonged period of time for the patient [38]

This study provided important and novel insights into the characteristics of patients who have been prescribed a sedative-hypnotic/anxiolytic from a primary care clinic and the patterns of documentation of its use. Several studies have aimed to characterize sedative-hypnotic/anxiolytic use in large samples using administrative data [7–10], however, such studies often do not have information on the direction of use, are susceptible to misclassification of indication of use, and do not have access to clinical notes that can provide further context to the prescribing and monitoring of these agents. This study attempted to address these gaps by using data based on patient EMR, which would include information on indication, dose and direction of medication use, and other monitoring parameters in the clinical notes, which are not accessible through most administrative claims data, and would not be subject to recall bias as would be the case for self-reported survey studies.

There are a few strengths and limitations of this study worth noting. While the design was enhanced by the inclusion of a random sample of 200 patients from four primary care clinics in Manitoba, Canada, medications are covered for all residents after an income-based deductible is met each year. As such, these findings may not be generalizable to other populations living in jurisdictions with a different health and drug coverage system. The EMR data studied extended previous work, by offering additional insights into EMR documentation in semi-structured categories, structured medication information and narrative encounter notes. However, we did not view specialist consultation records or hospital discharge summaries. Importantly, we did not confirm that prescriptions documented in the EMR were actually dispensed by a pharmacy. While encounter notes were reviewed for adverse events recorded after sedative-hypnotic/anxiolytic initiation, we are unable to determine with certainty if the sedative-hypnotic/anxiolytic actually caused the adverse event. Information not documented in the encounter notes does not necessarily mean the information was not communicated to the patient. However, this study has the potential to inform a standardized approach to documenting sedative-hypnotic/anxiolytic use in primary care. We included active patients between 2014 and 2016, and collected data over a four-month period in 2018. As a result, follow-up times may vary for each included patient.

Our study found many patients have used sedative-hypnotic/anxiolytic agents for greater than the recommended duration of use for anxiety or insomnia, and inconsistent documentation on tapering and monitoring of these agents. Standardized documentation of a monitoring plan that involves an assessment of the efficacy and safety of these agents at specific follow-up points, discussion of a tapering plan, and barriers and facilitators for carrying out a tapering plan has the potential to minimize the duration of use of these agents. Future research should examine strategies for effectively communicating risk versus benefit of these agents prior to prescribing and dispensing. Communicating expectations, rationale for short-term use, and a clear monitoring and tapering plan has been reported in previous literature to be important in engaging patients in shared decision-making about benzodiazepines [39, 40]. This study suggests inconsistent documentation of this information, but further clarification would be worthy of future study. This would especially be useful in light of practice standards from various jurisdictions regarding benzodiazepine prescribing and the need for a discontinuation strategy [41].

## Supplementary Information

**Additional file 1: Supplementary Table 1. **Data collection  form 
