# Supplementary material for: Insights into patient characteristics and documentation of the use of sedative-hypnotic/anxiolytics in primary care: a retrospective chart review study
Source: BMC Prim Care. 2022 May 10;23:111. doi: 10.1186/s12875-022-01724-9 (PMC9087974; doi:10.1186/s12875-022-01724-9)
Supplement: Supplementary file 1 — Additional file 1: Supplementary Table 1. Data collection form [file 12875_2022_1724_MOESM1_ESM.docx]

**Insights into Patient Characteristics and Documentation of the Use of Sedative-Hypnotic/Anxiolytics in Primary Care: A Retrospective Chart Review Study**

Kiana Gozda^1^, Joyce Leung^2^, Lindsay Baum^1^, Alexander Singer^3^, Gerald Konrad^3^, Diana E. McMillan^4^, Jamie Falk^1^, Leanne Kosowan^3^, Christine Leong^1,5^

^1^College of Pharmacy, Rady Faculty of Health Sciences, University of Manitoba

^2^Faculty of Science, University of Manitoba

^3^Department of Family Medicine, Max Rady College of Medicine, Rady Faculty of Health Sciences, University of Manitoba

^4^College of Nursing, Rady Faculty of Health Sciences, University of Manitoba

^5^Deparment of Psychiatry, Max Rady College of Medicine, Rady Faculty of Health Sciences, University of Manitoba

**Supplementary Table: Data Collection Form**

| Table 1: Phase 1 Project Indicators | | | |
| --- | --- | --- | --- |
| Variable Name | Description | Measure | Source |
| Patient Variable | | | |
| Sex | Patient sex | Female, Male, Undefined | Medical history band in EMR |
| Age | Patient date of birth | DATE (Jan – 01 – 2014) | Medical history band in EMR |
| Comorbidities | Type of comorbid condition requiring care while on sedative-hypnotic/anxiolytic medication | Diagnoses chronic conditions | medication band from the EMR |
| Co-medications | Other prescription medications taken while on sedative-hypnotic/anxiolytic medication | Type of medication | medication band from the EMR |
| Smoking status | Patent history of smoking status | Categorical (current, past, non-smoker, not recorded) | medical history band from EMR |
| Alcohol status | Patent history of alcohol status | Categorical (none, <7 week, 7-14 week, >14 week, not recorded) | medical history band from the EMR |
| Treatment Variables | | | |
| Sedative-hypnotic/anxiolytic | Patients current status of use | Categorical **(new** (≤1 year), **chronic** (>1), **past** (inactive for >1 yr)) | Medication band from the EMR |
| Indication of sedative-hypnotic/anxiolytic | Reason patient initiated sedative-hypnotic/anxiolytic | Categorical  (For sedative-hypnotic: Anxiety, sleep, spasticity, schizophrenia, cancer, palliative care, alcohol withdrawal, epilepsy, combination, other, undetermined) | Clinical notes from the EMR |
| Pattern of use | Patients pattern of medication use in the year prior to last prescription | Categorical (Acute (≤8 weeks), short term chronic (>8 to <3 months), intermediate chronic (≥3 months - <1 year), long-term chronic (≥1 year), intermittent (>1 month break between use), other) | Medication band from the EMR |
| Duration of use | Date of last prescription - date of first prescription | Categorical (≤1 year, >1-4 years ≥5 years) | Medication band from the EMR |
| Frequency of use | Daily frequency of patient use | Categorical (Daily, /Week, /month, unknown) | Medication band from the EMR |
| Medication dose | Maximum lorazepam or dose of z-drug (mg) per day of last prescription | Continuous (mg per day) | medication band from the EMR |
| Psychosocial intervention | Patient history of use of psychosocial intervention  Type of intervention | Dichotomous Y/N  Type | medical history band from the EMR |
| Tapering attempts | Patent tapering attempts since first prescription | Dichotomous Y/N | medical history band clinical notes from EMR |
| Past use of other central nervous depressant agents | Other prescription medications taken while on sedative-hypnotic/anxiolytic medication | Dimenhydrinate | medication band from the EMR |
| Social support and life stressor | Documentation of types of social support and presence of life stressors | Free-standing notes | Clinical notes from the EMR |
| Parameters for monitoring | Patient history of use of scales for monitoring: VAS, sleep scale, anxiety scale, mini mental status exam, withdrawal scale, and any other indicators of positive or negative outcomes of sedative-hypnotic/anxiolytic use | Name of scale  Score  Other Free-standing notes | medical history band and clinical notes from the EMR |
